# Increasing Coverage and Decreasing Inequity in Insecticide-Treated Bed Net Use among Rural Kenyan Children

**DOI:** 10.1371/journal.pmed.0040255

**Published:** 2007-08-21

**Authors:** Abdisalan M Noor, Abdinasir A Amin, Willis S Akhwale, Robert W Snow

**Affiliations:** 1 Malaria Public Health and Epidemiology Group, Centre for Geographic Medicine Research—Coast, Kenya Medical Research Institute/Wellcome Trust Research Programme, Nairobi, Kenya; 2 Division of Malaria Control, Ministry of Health, Nairobi, Kenya; 3 Centre for Tropical Medicine, University of Oxford, John Radcliffe Hospital, Oxford, United Kingdom; University of London, United Kingdom

## Abstract

**Background:**

Inexpensive and efficacious interventions that avert childhood deaths in sub-Saharan Africa have failed to reach effective coverage, especially among the poorest rural sectors. One particular example is insecticide-treated bed nets (ITNs). In this study, we present repeat observations of ITN coverage among rural Kenyan homesteads exposed at different times to a range of delivery models, and assess changes in coverage across socioeconomic groups.

**Methods and Findings:**

We undertook a study of annual changes in ITN coverage among a cohort of 3,700 children aged 0–4 y in four districts of Kenya (Bondo, Greater Kisii, Kwale, and Makueni) annually between 2004 and 2006. Cross-sectional surveys of ITN coverage were undertaken coincidentally with the incremental availability of commercial sector nets (2004), the introduction of heavily subsidized nets through clinics (2005), and the introduction of free mass distributed ITNs (2006). The changing prevalence of ITN coverage was examined with special reference to the degree of equity in each delivery approach. ITN coverage was only 7.1% in 2004 when the predominant source of nets was the commercial retail sector. By the end of 2005, following the expansion of heavily subsidized clinic distribution system, ITN coverage rose to 23.5%. In 2006 a large-scale mass distribution of ITNs was mounted providing nets free of charge to children, resulting in a dramatic increase in ITN coverage to 67.3%. With each subsequent survey socioeconomic inequity in net coverage sequentially decreased: 2004 (most poor [2.9%] versus least poor [15.6%]; concentration index 0.281); 2005 (most poor [17.5%] versus least poor [37.9%]; concentration index 0.131), and 2006 with near-perfect equality (most poor [66.3%] versus least poor [66.6%]; concentration index 0.000). The free mass distribution method achieved highest coverage among the poorest children, the highly subsidised clinic nets programme was marginally in favour of the least poor, and the commercial social marketing favoured the least poor.

**Conclusions:**

Rapid scaling up of ITN coverage among Africa's poorest rural children can be achieved through mass distribution campaigns. These efforts must form an important adjunct to regular, routine access to ITNs through clinics, and each complimentary approach should aim to make this intervention free to clients to ensure equitable access among those least able to afford even the cost of a heavily subsidized net.

## Abstract 2

Noor and colleagues found low levels of use of insecticide-treated mosquito nets when nets were mainly available through the commercial sector. Levels increased when subsidized nets were introduced and rose further when they were made available free.

## Editors' Summary

**Background.:**

Malaria is one of the world's most important killer diseases. There are over a million deaths from malaria every year, most of those who die are children in Africa. Frequent attacks of the disease have severe consequences for the health of many millions more. The parasite that causes malaria is spread by bites from certain species of mosquito. They mostly bite during the hours of darkness, so sleeping under a mosquito net provides some protection. In some countries where malaria is a problem, bed nets are already used by many people. A very much higher level of protection is obtained, however, by sleeping under a mosquito net that has been impregnated with insecticide. The insecticides used are of extremely low toxicity for humans. As insecticide-treated nets (ITNs) are a relatively new idea, people do need to be persuaded to buy and use them. ITNs must also be re-impregnated regularly, although long-lasting ones that remain effective for 3–5 y (or 21 washes) are now widely distributed. The nets are inexpensive by Western standards but the people who are most at risk of malaria have very little income. Governments and health agencies are keen to increase the use of nets, particularly for children and pregnant women. The main approach used has been that of “social marketing.” In other words, advertising campaigns promote the use of nets, and their local manufacture is encouraged. The nets are then sold on the open market, sometimes with government subsidies. This approach has been very controversial. Many people have argued that ways must be found to make nets available free to all who need them, but others believe that this is not necessary and that high rates of ITN use can be brought about by social marketing alone.

**Why Was This Study Done?:**

It has been known for more than ten years that ITNs are very effective in reducing cases of malaria, but there is still a long way to go before every child at risk sleeps under an ITN. In Kenya, a country where malaria is very common, a program to increase net use began in 2002, using the social marketing approach. In 2004 most of the nets available in Kenya were those on sale commercially. In October 2004 health clinics started to distribute more heavily subsidized ITNs for children and pregnant women and, in 2006, a mass distribution program began of free nets for children. The researchers, based at the Kenya Medical Research Institute (KEMRI), wanted to find whether the number of children sleeping under ITNs changed as a result of these changes in policy. They also wanted to see how the rate of net use varied between families of different socioeconomic levels, as the poorest children are known to be most likely to die from malaria.

**What Did the Researchers Do and Find?:**

This is a large study involving 3,700 children in four districts of Kenya. The researchers conducted surveys and then calculated the rates of net use in 2004, 2005, and 2006. In the first survey, when nets were available to most people only through the commercial sector, only 7% of children were sleeping under ITNs, with a very big difference between the poorest families (3%) and the least poor (16%). By the end of 2005, the year in which subsidized nets became increasingly available in clinics, the overall rate of use rose to 24%. By the end of 2006, following the free distribution campaign, it was 66%. The 2006 figure was almost exactly the same for the poorest and least poor families.

**What Do These Findings Mean?:**

The rate of net use in the districts in the survey is much higher than expected, even though one-third of children were still not protected by ITNs. The sharp increases—particularly among the poorest children—after heavily subsidized nets were introduced and then after the free mass distribution suggests that this is a very good use of the limited amount of funds available for health care in Kenya and other countries where malaria is common. If fewer Kenyan children have malaria there will be cost savings to the health services. While some might claim that it is obvious that nets will be more widely used if they are free, there has been heated debate as to whether this is really true. Evidence has been needed and this research now provides strong support for free distribution. The study has also identified other factors which will be important in the continuing efforts to increase ITN use.

**Additional Information.:**

Please access these Web sites via the online version of this summary at http://dx.doi.org/10.1371/journal.pmed.0040255.

- The US Centers for Disease Control and Prevention provide information on malaria and on insecticide-treated nets (in English and Spanish)
- The MedlinePlus encyclopedia contains a page on malaria (in English and Spanish). MedlinePlus brings together authoritative information from the US National Library of Medicine, National Institutes of Health, and other government agencies and health-related organizations
- Information is available from the World Health Organization on malaria (in English, Spanish, French, Russian, Arabic, and Chinese) and from the Roll Back Malaria Partnership on the use of insecticide-treated nets
- For information about the Medical Research Institute see the organization's Web site
- The BBC Web site has a “country profile” about Kenya
- Malaria data and related publications can be found on the Malaria Atlas Project Web site, which is funded by the Wellcome Trust, UK and is a joint project between the Malaria Public Health & Epidemiology Group, Centre for Geographic Medicine, Kenya and the Spatial Ecology & Epidemiology Group, University of Oxford, UK
- The Kenya Ministry of Health, Division of Malaria Control Web site has useful information on malaria epidemiology and policies for Kenya

## Introduction

The gulf between levels of childhood mortality in sub-Saharan Africa and access to simple, cost-effective interventions known to significantly reduce mortality is immoral [1]. For over ten years it has been known that insecticide-treated bed nets (ITNs) can reduce childhood mortality by 17% [2]. In 1998 the Roll Back Malaria (RBM) movement was launched with one of its primary objectives to increase ITN coverage among vulnerable groups, such as children and pregnant women, to over 60% [3]. RBM has recently revised this ITN objective to reach 80% coverage by 2010 [4]. This change in target followed the RBM publication of the current status of ITN coverage in Africa as part of its World Malaria Report [5]. Of 34 malaria-endemic countries in Africa providing recent national data, only one (Eritrea) had achieved ITN coverage among children aged less than 5 y of more than 60% [5]. Reasons for this dismal progress has been the subject of much debate [6–9] and largely centre around divergent views on optimal strategies to deliver ITNs to economically and biologically vulnerable groups across Africa.

During the early days of RBM, the technical advice to countries provided by World Health Organization (WHO) was to create an “enabling environment” that allowed malaria-endemic countries to embrace multiple approaches to providing ITNs [10]. These approaches included building sustainable private for-profit markets, for example through the NETMARK initiative in nine African countries [11]; creating a not-for-profit commercial sector through social marketing, a model promoted by Population Services International (PSI) operating in 23 African countries [12]; or the less popular at the time option of providing ITNs free of charge through clinics, or as first suggested in 1995, through vaccine campaigns [13].

The best-practice debate has been driven by personal opinion [6,7] or data on temporal changes in ITN coverage associated with single delivery approaches [14–16]. Where data exist they compare information on a single delivery model against a baseline without significant intervention or iterative increases in net coverage associated with a single, nationally adopted approach to delivery. Rarely is it possible to compare incremental coverage associated with different delivery models. Here we present a serial observation of ITN coverage among rural Kenyan communities exposed at different times to the range of delivery models each with legitimate claims to improve ITN access. Our emphasis throughout this study was to examine how ITN access by the poorest sectors of rural communities might best be achieved with each approach.

## Methods

### The Kenya ITN Context

Prior to the launch of Kenya's National Malaria Strategy in April 2001 [17], access to nets was limited to the private for-profit retail sector and special project-based distributions through research- or nongovernmental organization-led community development initiatives [18]. In 2000 the Kenya Ministry of Health (MoH) developed with partners an ITN strategy paper [19] that attempted to accommodate two competing views on ways to reach the government's target of 60% coverage of populations at risk by 2005. The first approach included ways to ensure that the ITN market is self-sustaining in the absence of long-term donor support by expanding the private sector through social marketing; the second approach, principally favoured by the MoH, was to provide ITNs free of charge to pregnant women and children under the age of 5 y to achieve rapid scale-up.

In January 2002 the UK Department for International Development (DFID) awarded PSI-Kenya US$33 million over 5 y to socially market partially subsidised ITN within the existing retail sector. The programme, named PSI Coverage Plus, was the only major operational ITN distribution initiative between 2002 and 2004 and aimed to target urban and rural retail outlets with Supanet ITNs across all malaria-endemic districts in Kenya. A two-tier pricing system of 350 Kenya Shillings (KES) (equivalent to US$4.7) in urban settings versus KES100 (US$1.3) in rural settings was implemented. The programme's aims were to increase community awareness of the value of ITNs thus creating a “net culture”; force existing retail prices down; and increase clients' willingness to pay for nets through a sustainable, unsubsidised commercial market [20].

In June 2004, DFID approved an additional US$19 million to PSI to establish a parallel distribution system of heavily subsidised ITNs to children and pregnant women through Maternal and Child Health (MCH) clinics, recognizing that these vulnerable groups might not be able to access socially marketed commercial sector nets. The programme began in October 2004, and during the first 6 mo Supanet ITNs were bundled with separate Powertab net treatment tablets (for every 6 mo) and distributed to MCH attendees. In May 2005 an additional US$37 million was committed by DFID to PSI to procure and distribute Supanet-branded long-lasting insecticidal nets (LLINs), Olyset and Permanet. These public sector nets were heavily subsidized pretreated nets (KES50; US$0.7) and branded with the MoH logo [20].

In February 2002 the MoH responded to the first call of the Global Fund to Fight AIDS, TB and Malaria (GFATM) for funding applications to secure five million nets and net treatments to provide free of charge to children under the age of 5 y and pregnant women. This application was unsuccessful. During round four of the GFATM awards in April 2004, Kenya's application was successful and US$17 million was approved to procure and distribute 3.4 million LLINs (Olyset and Permanet brands) free of charge to children under the age of 5 y. This represented, at the time, the largest successful award for free distribution of LLINs in Africa. The implementation of the free mass distribution of LLINs was arranged in two phases during 2006. During the first phase, 21 of Kenya's 70 districts were selected for distribution of LLINs from 8 to 12 July 2006 and integrated with the national measles catch-up vaccination campaign. Health facilities and centralised non-health facility posts were identified by the Kenya Expanded Programme on Immunisation and used as delivery points of both measles vaccine and LLINs to each child under the age of 5 y. A second mass distribution of LLINs, not integrated with any other intervention, took place from 25 to 27 September 2006 in 24 additional districts using previous mass vaccine campaign delivery centres as distribution points.

### Study Area

The study was carried out in four districts purposively sampled in collaboration with the MoH to provide detailed longitudinal milestone data on changing access to interventions proposed within the Kenya National Malaria Strategy between 2001 and 2006 [21,22]. The study districts represent the range of dominant malaria epidemiological situations that prevail across Kenya: Kwale on the coast with seasonal high-intensity malaria transmission; Bondo on the shores of Lake Victoria with perennial high-intensity transmission; Greater Kisii district (combining the new districts of Kisii Central and Gucha) with seasonal low transmission conditions of the Western highlands; and Makueni district, a semi-arid area with acutely seasonal low malaria transmission. Between 63% and 71% of households in the rural areas of each of the four districts were living below the poverty line (equivalent to US$1 per day) in 1999, compared to the national average of 54% [23]. The districts were also representative of rural districts in Kenya with respect to net delivery since 2001, with service providers including the full-cost commercial and social marketing retail sector; a research team in parts of Bondo district [24]; nongovernmental organization delivery to selected communities in Greater Kisii (Merlin and World Vision) and Kwale (Plan International and The Aga Khan Foundation); time-limited MoH provision of free nets to pregnant women in 2001 in all districts [25] and to children and pregnant women in Bondo and Gucha districts in 2005 [26]; and subsidized PSI clinic distribution since October 2004 and mass, free distribution in 2006 across all districts.

Within each district, rural enumeration area (EA) boundaries were digitized with ARCGIS 9.0 (ESRI, http://www.esri.com/) and each polygon attributed to population totals derived from the last national census in 1999 [27]. A sample of 18 rural EA polygons, covering approximately 6,500 people per district, was randomly selected from each district to form the basis of the longitudinal community surveillance. Following community sensitisation, all homesteads within an EA polygon were mapped and heads of homesteads were given the purpose of the longitudinal study and asked whether they wished to participate. All de jure resident homestead members were enumerated, including details of date of birth and sex, and issued a unique identifier for follow-up.

### The Longitudinal Cohort

During December/January of 2004/5, 2005/6, and 2006/7, just after the short rains, a cohort of children under the age of 5 y was established to track, by interviewing mothers or caretakers, the ownership and use of bed nets, including details on the net brand, where and when they were obtained, and whether nets had been treated with an insecticide during the previous 6 mo. Interviewers were instructed to observe the nets and record details of the colour, imprinted logos, and shape of the net to match the net types delivered by different partners in each district at different times. All children resident in 2004 were recruited into the cohort and exited during subsequent census rounds if they had out-migrated, homesteads or guardians subsequently refused participation, they had reached their fifth birthday, or they had died. New children were recruited into the cohort if they had migrated into the homestead between census rounds or were identified through detailed birth histories of all resident women aged 15–49 y as having been born during the interval. New infants who did not survive the interval between census rounds were included in the cohort. In-migrations that out-migrated between the census rounds were not included in the cohort and were regarded as short-term visitors not permanently resident.

During the 2005/6 annual census round, representing the reference midpoint of the surveillance period, details were recorded on each homestead relating to key asset indicators, including: homestead head education level and occupation; housing characteristics (type of wall, roof, and floor); source of drinking water; type of sanitation facility; homestead size; and persons per sleeping room (see Table S1).

### Data Entry and Analysis

Data entry and storage were undertaken using Microsoft Access, and analysis was undertaken using STATA version 9.2 (Stata, http://www.stata.com) and ARCGIS 9.0 (ESRI). All information specific to the EA, homestead, and mother or guardian were linked to the relevant child through the use of a primary homestead identifier consistent across all data sets. To account for unequal probabilities of selection of EAs, all results were weighted (weight = 1/probability of selection) and precision of proportions (95% confidence intervals [CIs]) were adjusted for clustering with EA as the primary sampling unit. A χ^2^ test was performed to compare net use proportions across subgroups within and between survey years. For comparisons of socioeconomic groups within a survey year, the Pearson χ^2^ statistic, accounting for clustering, was used. This statistic is turned into an F-statistic using the second-order Rao and Scott correction and *p*-values interpreted the same way as the Pearson χ^2^ statistic for data without clustering [28,29].

A homestead wealth index was constructed from the asset indicators using principal component analysis. Weights (scoring coefficients) derived from the first principal component were used to construct the wealth index [30]. Weights for each asset indicator from the first principal component were then applied to each homestead record to produce a wealth index. Wealth asset indices were developed separately for each district to allow for innate differences in the meaning of different assets between districts. Each homestead was then assigned to a district-specific wealth quintile. Net ownership by children in the cohort was examined serially and by source according to wealth asset quintiles. Inequity in net coverage over time and by source was analysed using the concentration index, which gives values between −1 and 1, with a value 0 indicating an absence of wealth-related inequality in net use among children [31]. Because net use is a “good” health variable, a positive value of the index indicates net use is concentrated among the wealthy. The concentration curve was plotted to illustrate changes in wealth-related inequality [31].

### Ethical Approval

Ethical approval was provided by the National Ethical Review Board IRB (Kenya Medical Research Institute SSC number 906).

## Results

A total of 2,761 homesteads were selected across the 72 rural communities located in the four districts in 2004. Three homesteads refused participation in 2004 (0.01%); 11,050 observations of children under the age of 5 y were made across the three survey years in 2004/5, 2005/6, and 2006/7. Over the three years, 155 (1.4%) usually resident children were visiting elsewhere at the time of the annual surveys, 66 (0.6%) children's parents or guardians refused to be interviewed and 133 (1.2%) children died before the census rounds in 2005/6 and 2006/7.

In 2004/5 the weighted proportion of children usually using a net, adjusted for clustering, was 13.9%, similar to the proportions of children using a net the night before the survey (Table 1). The small difference between usual use of a net and net use the night before survey was a consistent finding across all surveys (Table 1). We elected to restrict subsequent analysis to net use the night before survey. In 2004/5 the majority of children (65%) who slept under a net were sleeping under nets purchased from the commercial retail sector, only 7% of children slept the night before the survey under nets treated with an insecticide within the last 6 mo, and the proportion ITN use among children living in the poorest quintile homesteads was one-fifth (2.9%) to the proportion of those living in the least poor homesteads (Table 1).

**Table 1:**
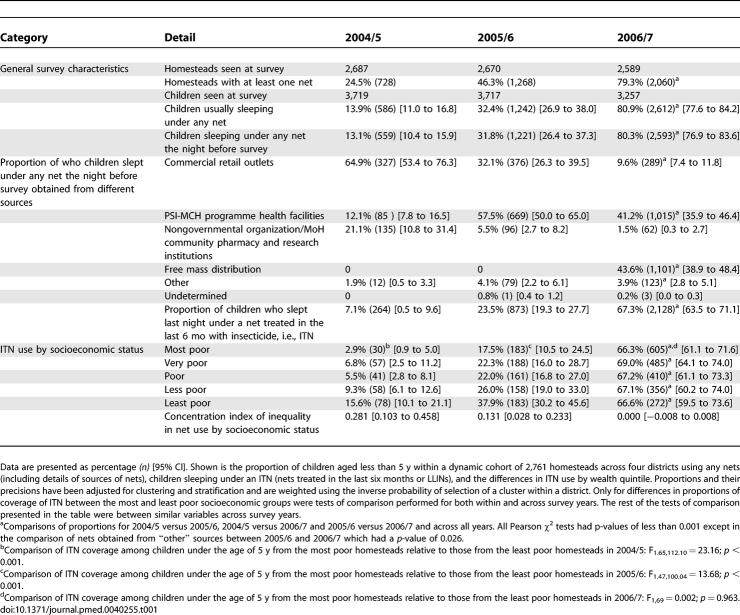
Net Usage by Children across Four Districts in Three Consecutive Survey Years

In December/January 2005/6, 12 mo after the baseline survey, the proportions of children sleeping under a net had increased to 32% where the dominant net source was the heavily subsidized PSI-MCH clinic nets; 58% of children using a net were using nets from this source (Table 1). The proportion of children sleeping under a net treated with an insecticide in the last 6 mo had tripled to over 23% compared to 2004/5 (*p* < 0.001). Children living in the poorest homesteads had proportionately the largest (six-fold) increase in ITN use over the 12 mo interval, rising from 2.9% in 2004/5 to 17.5% in 2005/6 (*p* < 0.001); however, children living in the least poor homesteads were still twice as likely to have slept under an ITN the previous night as those in the poorest homesteads (Table 1). These results are reinforced by the concentration indices, which remained positive (net use highest among the wealthier groups) in both rounds of the survey, although the measure of wealth-related inequality, the concentration index, fell from 0.281 in 2004/5 to 0.131 in 2005/6.

**Figure 1:**
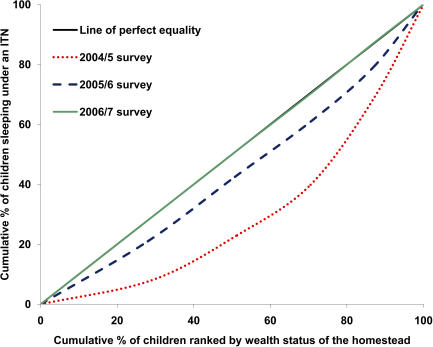
Degree of Inequality in Children Sleeping under an ITN in 2004/5, 2005/6, and 2006/7 in Homesteads of Different Wealth Status in the Four Districts in Kenya The concentration curve below the line of perfect equality indicates that ITN use is concentrated among higher socioeconomic groups. When the curve is coincident on the line of perfect equality, then there is no wealth-related inequality in ITN use.

**Figure 2:**
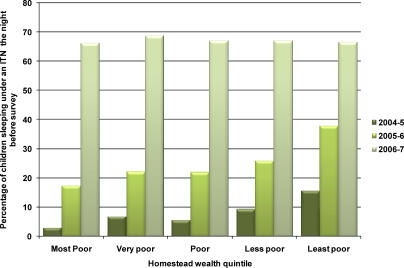
Proportion of Children Sleeping under an ITN in 2004/5, 2005/6, and 2006/7 in Homesteads of Different Wealth Status in Four Districts in Kenya

By December/January 2006/7, the proportion of children sleeping under a net increased to 81%, with the two dominant sources of nets being the free mass campaign (44%) and the PSI-MCH clinics (41%) (Table 1). Within 12 mo of the previous survey, the proportion of children sleeping under an ITN had doubled to 67%; 44% of children were sleeping under an ITN provided during the mass campaign. The largest increase in the proportion of children sleeping under an ITN was among those from homesteads in the poorest quintile, from 17.5% in 2005/6 to 66.3% in 2006/7. There was no statistically significant difference in the proportion of children using an ITN between highest and lowest wealth quintiles in 2006/7 (*p* = 0.963). The poverty concentration index declined from 0.131 in 2005/6 to 0.000 in 2006/7 (Table 1). The concentration curve indicated, for the first time, the absence of wealth-related inequality in net use (Figure 1) further illustrated by the graph of actual proportions (Figure 2).

**Figure 3:**
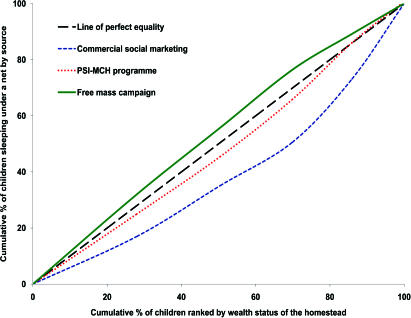
Degree of Inequality in Socioeconomic Targeting by the Three Principal Net Delivery Mechanisms in Four Districts in Kenya by 2006/7 Delivery mechanisms included commercial social marketing, the PSI-MCH programme, and a free mass campaign.

By the end of September 2006, the three principal net distribution strategies (retail social marketing, heavily subsidized clinic distribution, and free mass distribution) were all operating in parallel, providing an opportunity to examine socioeconomic targeting of each of the delivery mechanisms (Table 2). In 2006/7 2.4% and 24.3% of children from the poorest homesteads slept under a net from the retail sector and the PSI-MCH programme, respectively, compared to 6.4% and 30.8% from the least poor. Conversely, by 2006/7 the highest proportion of children from the poorest homesteads slept under nets from the free mass campaign (most poor/least poor: 36.6%/25.8%). This pattern is further illustrated by the concentration curve (Figure 3), which shows that the free mass campaign is the only delivery channel favouring the poorest children; the PSI-MCH programme was marginally in favour of the least poor, and the commercial sector was the most inequitable in favour of the least poor.

**Table 2:**
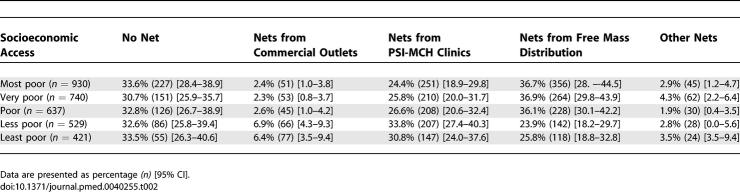
Variations in Children's Net Use the Night before the 2006/7 Survey by Source across Different Socioeconomic Groups

## Discussion

We have shown that a concerted, multi-pronged approach to ITN delivery in rural areas of Kenya over 3 y resulted in over 60% of children sleeping under a net treated with insecticide, surpassing the original RBM target set in Abuja in 2000 [3]. However, at the end of 2004 this target seemed almost unattainable, with only 7% of rural children reported to be sleeping under an ITN and only 3% among the poorest sectors of these communities. Radical changes in bilateral support to PSI to adapt their social marketing strategy to included MCH clinics resulted in important changes in ITN coverage (24%) by the end of 2005, but coverage still favoured the least poor children. The most dramatic increases in ITN coverage were seen during the last year of the surveillance period at the end of 2006. Through two single mass campaigns in July and September 2006, coverage of ITN use rose to over 67% and was particularly successful at reaching the poorest children (Tables 1 and 2). The concentration index of ITN coverage, which is a measure of inequality, decreased from 0.281 in 2004/5 to 0.000 in 2006/7, indicating an absence of inequality in net use (Figures 1 and 2; Table 1) coincidental with the expansion in different mechanisms of delivery.

In recent years, there has been a consensus among national ministries of health, development partners, and other stakeholders that access to health interventions should be made pro-poor [30]. Although gaps in ITN coverage between the least- and most-poor groups have been declining elsewhere in Africa [5], the most-poor remain the least well covered. Our results show that ITN coverage among children was similar across all wealth groups by 2006/7. and nets provided through the free mass campaign actually preferentially covered children from the poorest-quintile homesteads while the heavily subsidised PSI-MCH clinic nets was considerably more equitable than the commercial social marketing (Figure 3; Table 2). Elsewhere in Africa pro-poor ITN interventions have been reported [32–35], but these efforts have been relatively small in scale. To the best of our knowledge this is the first time in Africa that a large-scale public health intervention, covering millions of people, has preferentially reached the most-poor quintiles of a community when compared to the least poor.

Unlike single cross-sectional surveys of ITN coverage, the value of the present study is its longitudinal observations among the same homesteads exposed at different times to different systems of delivery. Such data help inform debates on whether ITN delivery should be free or subsidized and whether they should be provided through routine clinics, mass campaigns, or the private sector. Our data clearly show that the most effective means to rapidly scale up ITN coverage, particularly among the poor, is through targeted free distributions, preferably coincidental with other campaigns such as mass vaccination. However, it does not necessarily follow that this should represent the only mechanism by which treated nets are distributed to rural communities.

The PSI programme of heavily subsidized net delivery through over 3,000 clinics since October 2004 has reported over 5 million sales of nets [20]. The complex distribution, ordering, and logistics of net commodities were run efficiently by an externally funded and managed system with the infrastructure and staff provided by the government and mission sectors [36]. Although there are no data to support the claim that the provision of subsidized nets at clinics increased use of health facilities, this may have occurred and is a similar argument used to increase attendance at mass Expanded Programme for Immunisation vaccine campaigns that provide free nets [15]. The PSI programme, however, did not reach nationally or internationally agreed targets of ITN coverage within the specified timelines. Similarly routine Expanded Programme for Immunisation services, provided free at clinics, often fail to reach their anticipated coverage targets [37]. We would argue that the constant availability of ITNs at clinics provides an essential early entry point for net use, particularly among young pregnant women who would otherwise not have access to ITNs if restricted to a single annual event targeting children as part of a mass distribution campaign. For vaccine-based programmes to retain effective herd immunity, periodic catch-up campaigns of single mass coverage of vulnerable populations are necessary. We would view this as an analogous position for effective ITN coverage. It is therefore not a question of choosing between the strategies but how to effectively combine them. Some sectors of our rural communities can afford subsidized ITNs, but when only these nets are available the most-poor access them least. It is likely that those bearing the brunt of the malaria burden in rural areas are those most distal to health services and the poorest. Provision of free nets would benefit these communities most. Our results are far less supportive of international donor-promoted efforts to expand the commercialization of ITN distribution in Africa [8,11]. This approach clearly failed to reach those most in need in Kenya, and despite aggressive marketing campaigns, and development of branded products and branded outlets, the incremental gains in ITN coverage were minimal.

To effectively integrate routine versus mass campaign ITN distribution requires careful planning. It is important that when planning integrated free mass campaigns, each component of the package is not jeopardized by the other, for example delaying immunization to meet the needs of ITN distribution. In Kenya, funding for the donor-supported PSI programme ends in 2007. Funds are available from the GFATM and the World Bank Booster Programme during 2007 and 2008 to continue LLIN distribution through mass campaigns. Replacing or “permanently” treating existing non-LLINs, covering future vulnerable pregnant women and their infants and expanding to pockets of the country where coverage has remained low all require long-term sustainable financing planned against projected spatially defined needs. These combined approaches require long-term evaluations, modelled on the approach presented, to monitor the synergies between delivery strategies to make sure they retain the equity required to reach those most in need. Whether funding for combined models of delivery comes from national budgets, direct budgetary support from donors, or through mechanisms such as the GFATM, Presidents Malaria Initiative, or the World Bank is beyond the scope of this paper. What we can say is that if funding is not secured for clinic supply and catch-up mass campaigns for LLIN delivery beyond 2008 the impressive, rapid progress toward the RBM target of 80% coverage by 2010 in Kenya will be lost.

## Supporting Information

Table S1 Asset Indicators and Their Weights Computed Using Principal Component Analysis to Construct Homestead Wealth Quintile Rankings for Each District (45 KB DOC)

## Abbreviations

CI: confidence interval
DFID: Department for International Development, UK Government
EA: enumeration area
EPI: Expanded Programme for Immunisation
GFATM: Global Fund to Fight AIDS, TB and Malaria
ITN: insecticide-treated bed net
LLIN: long-lasting insecticidal net
MCH: Maternal and Child Health
MoH: Ministry of Health (Kenya)
PSI: Population Services International
RBM: Roll Back Malaria initiative
WHO: World Health Organization.
