# Supplementary material for: Increasing Coverage and Decreasing Inequity in Insecticide-Treated Bed Net Use among Rural Kenyan Children
Source: PLoS Med. 2007 Aug 21;4(8):e255. doi: 10.1371/journal.pmed.0040255 (PMC1949846; doi:10.1371/journal.pmed.0040255)
Supplement: Supplementary file 1 [file pmed.0040255.st001.doc]

**Supporting Information B:**

**Constructing homestead wealth indices from asset indicators**

In constructing asset-based wealth indices, principal component analysis (PCA) is regarded one of the best methods to use and has become widely relied upon over the past decade (Filmer & Pritchett 2001; Hulme & McKay 2005; Noor et al., 2006). When using PCA, the scoring coefficients (weights assigned to each variable) of the first component are typically used to represent the wealth index (Filmer & Pritchett 2001). The table here summarises the proportion of variance explained by the first component and the resulting variable weights by district. The PCA was performed by districts to take account of the innate but important contribution to well-being of similar variables across different settings.

|  | **District (percentage variance explained by the first principal component)** | | | |
| --- | --- | --- | --- | --- |
| **Variable** | **Bondo**  **(15.1%)** | **Greater Kisii (19.7%)** | **Kwale**  **(21.5%)** | **Makueni**  **(17.1%)** |
| Homestead size* | -0.0048 | -0.1647 | -0.1435 | -0.1958 |
| Persons per sleeping room* | -0.1076 | -0.1372 | -0.1696 | -0.2883 |
| **Homestead head education level** |  |  |  |  |
| No education | -0.1580 | -0.2317 | -0.3073 | -0.1856 |
| Primary incomplete | -0.0917 | -0.1387 | 0.0805 | -0.1237 |
| Primary complete | 0.0367 | -0.0153 | 0.0996 | -0.0177 |
| Secondary and above | 0.2681 | 0.3754 | 0.2609 | 0.3426 |
| **Homestead head occupation** |  |  |  |  |
| Works for pay | 0.0962 | 0.3788 | 0.2681 | 0.3405 |
| Receives income from spouse or family member | -0.0089 | -0.2254 | -0.2621 | -0.3116 |
| Works upaid on family farm or business | -0.0616 | -0.0795 | 0.0614 | 0.0144 |
| Unemployed | -0.0265 | -0.0780 | -0.0437 | -0.0812 |
| **Housing structure and access to water and sanitation** |  |  |  |  |
| Ownership flush toilet or pit latrine | 0.4075 | 0.0475 | 0.3253 | 0.1754 |
| House with stone, brick or concrete wall | 0.4945 | 0.4929 | 0.3562 | 0.3787 |
| House with cement, tile, linoleum or wood floor | 0.4800 | 0.4979 | 0.3324 | 0.3964 |
| House with grass/thatch roof | -0.2970 | -0.1747 | -0.3221 | -0.3960 |
| Homestead uses piped or borehole water | 0.0142 | 0.1256 | 0.2966 | -0.0065 |
| Homestead uses rain water or from streams and rivers | 0.2672 | NA | -0.0268 | -0.0495 |
| Homestead uses water from ponds or dams | -0.2592 | NA | -0.3030 | 0.0850 |

* These are continuous variables. All other variables have a yes/no response

NA- refers to variables that were dropped due to zero variance.

**Note:** The negative contribution of homestead size and persons per room to the index follows from the summaries of the data, which, on average, showed that larger families generally had lower quality asset indicators including fewer number of sleeping rooms.

**References**

Filmer D, Pritchett LH (2001) Estimating wealth effects without expenditure data – or tears: an application to educational enrolments in states of India. Demography **38**: 115-132.

Hulme D, McKay A (2005) Identifying and Measuring Chronic Poverty: Beyond Monetary Measures. The Many Dimensions of Poverty, International Conference, Brasilia, Brazil, August 2005.

Noor AM, Omumbo JA, Amin AA, Zurovac D, Snow RW (2006). Wealth, mother's education and physical access as determinants of retail sector net use in rural Kenya. Malaria J **5**; 5.
